# Bactericidal Activity of Graphene Oxide Tests for Selected Microorganisms

**DOI:** 10.3390/ma16114199

**Published:** 2023-06-05

**Authors:** Katarzyna Olczak, Witold Jakubowski, Witold Szymański

**Affiliations:** 1 Department of Endodontics, Medical University of Lodz, Pomorska 251, 92-213 Lodz, Poland; 2 Division of Biophysics, Institute of Materials Science and Engineering, Lodz University of Technology, ul Stefanowskiego 1/15, 90-924 Lodz, Poland; witold.jakubowski@p.lodz.pl (W.J.); witold.szymanski@p.lodz.pl (W.S.)

**Keywords:** antibacterial activity, dentistry, graphene, graphene oxide, medicine

## Abstract

The aim of this study was to determine the bactericidal potential of graphene oxide (GO) in contact with four species of bacteria: *E. coli*, *S. mutans*, *S. aureus* and *E. faecalis*. Bacterial cell suspensions of each species were incubated in a medium containing GO, with incubation times of 5, 10, 30 and 60 min, at final concentrations of 50, 100, 200, 300 and 500 μg/mL. The cytotoxicity of GO was evaluated using live/dead staining. The results were recorded using a BD Accuri C6 flow cytofluorimeter. Obtained data were analyzed using BD CSampler software. A significant bacteria viability reduction was noted in all GO-containing samples. The antibacterial properties of GO were strongly influenced by GO concentration and incubation time. The highest bactericidal activity was observed at concentrations of 300 and 500 μg/mL for all incubation times (5, 10, 30 and 60 min). The highest antimicrobial potential was observed for *E. coli*: after 60 min, the mortality rate was 94% at 300 µg/mL GO and 96% at 500 µg/mL GO; the lowest was found for *S. aureus*—49% (300 µg/mL) and 55% (500 µg/mL).

## 1. Introduction

Graphene is an allotropic variety of carbon with a hexagonal arrangement of atoms resembling a honeycomb. As it is only one atom thick, it is therefore assumed to be a two-dimensional structure. Graphene has enjoyed considerable popularity in the world of science and industry, and more recently in medicine. The first theoretical work on it appeared as early as 1947, and its structure was first described in 1962 by Hanns-Peter Boehm, using X-ray diffraction.

Graphene is produced using several techniques, each of which allows for different potential applications in science and industry. The original method of obtaining graphene involving mechanical exfoliation, i.e., the peeling off of individual small transverse planes of graphite (graphene) from graphite using adhesive tape, is only used for purely research applications; however, the product has excellent properties such as low structure deformation. The production of large-area graphene involves other production methods, e.g., chemical vapor deposition (CVD) on thin metal films such as Cu [1,2] or epitaxial growth on silicon carbide growth substrates [3]. Alternatively, high-quality large-area graphene can be obtained using the HSMG method [4].

Graphene derivatives such as graphene oxide and reduced graphene oxide have also been identified [5,6]; however, the former is of greatest interest for biomedical applications [7,8]. Graphene oxide is an oxidized form of graphene in which numerous oxygen functional groups are attached to the carbon plane. It consists of carbon flakes of various transverse sizes and a thickness of approximately 1.1 ± 0.2, with functional groups present on both sides of the flake and at its edges [9]. Graphene oxide is produced from graphite, which is converted to graphite oxide by oxidation. It can be obtained using methods such as the Hummers method using strong oxidizers such as H_2_SO_4_, KNO_3_ and KMnO_4_. The oxidized graphite (intercalated with oxygen groups) is then subjected to ultrasound treatment to delaminate it (exfoliation) and transform it into graphene oxide [10]. GO can also be obtained from carbon nanotubes by cutting them into graphene ribbons (graphene oxide nanoribbons (GONRs)) [11].

Due to the presence of oxygen functional groups, GO is hydrophilic and mixes well with water to form time-stable colloidal solutions. It also demonstrates poor current conductivity due to the oxygen groups changing the hybridization of the carbon atoms from sp^2^ to sp^3^. However, it should be noted that the parameters of GO can differ depending on the production method.

For many years, there has been a growing need for agents to combat infections, particularly considering the global rise in drug and antibiotic resistance in many strains of bacteria and fungi. Since the first papers on the bactericidal activity of graphene and its derivatives were published, hopes have been raised for its use in various branches of medicine.

In the case of GO, the mechanism of antibacterial action remains unclear. However, ongoing experimental studies have indicated several distinct patterns of biocidal interaction.

It is widely accepted that GO damages the bacterial cell membrane. A transmission electron microscopy (TEM) study found that when placed in contact with *E. coli* bacteria, GO nanosheets cause the removal of phospholipids from their cell membranes [12]. This has been confirmed for both Gram-negative *E. coli* and Gram-positive *S. aureus* bacteria [13]. Interestingly, Zou et al. attribute the destruction of the cell membrane to a mechanism based on micro-vibrations of the nanosheets, which thus pierce the membrane; this action led to the nanosheets being termed *nanoknives* by these authors [14]. Alternatively, it has been proposed that GO can increase oxidative stress on bacteria, leading to the oxidation of lipids, nucleic acids and proteins, resulting in cell membrane destruction and cell growth inhibition [1,15,16].

Alternatively, studies suggest that bacterial proliferation may be inhibited by the GO “wrapping” around bacterial cells [17,18]. In such cases, two sets of results have been identified: in the first, metabolic activity is inhibited without any apparent damage to the cell membrane, while in the second, the main cause of bacterial death remains membrane depolarization leading to metabolic disruption of bacterial cells. Finally, it is possible that GO may be converted into a much more toxic form, rGO, found to act against the marine bacteria of the genus Shewanella; the authors term this mechanism the self-killing effect [19,20].

Regardless of which mechanism of bactericidal action of graphene oxide we accept as the leading hypothesis, or whether we assume that all mechanisms may act simultaneously, the nature of this interaction appears to be unencumbered by one of the main current problems in the treatment of bacterial infections, i.e., increasing antibiotic resistance among pathogenic bacterial strains. The physical interaction of GO on cell membranes or the interaction disrupting normal bacterial metabolism could be of great importance in the applications of GO and other graphene derivatives against infections with strains with increased drug resistance [21].

In the literature, there are many experiments showing the high bactericidal effectiveness of GO against various types of bacteria. In their study, Di Giulio et al. showed a significant growth inhibition of *S. aureus* at 2 and 24 h and *P. aeruginosa* at 2 h after incubation with graphene oxide [22]. Another study showed a significant reduction in the viability of *S. aureus* at 12 h and *E. coli* at 168 h [21]. Promising results were shown by F. Zou et al. Their study on bacterial strains of *M. smegmatis*, *E. coli* and *S. aureus* revealed a 99% loss of viability in all observed species after only 15 min of incubation with GO [23]. Ghanim et al. noted a GO concentration-dependent increase in the inhibition zone for *E. coli* and *S. aureus*. Bactericidal effectiveness measured by the zone of inhibition was higher for *S. aureus* [24]. GO has also been shown to be effective against *E. faecalis* and *S. mutans* [22,25,26]. Both mentioned bacterial strains are responsible for oral infections, and *E. faecalis* is also responsible for serious systemic infections [27,28].

It seems particularly important to demonstrate that graphene oxide will demonstrate a bactericidal effect against the entire spectrum of pathogenic bacteria and, importantly, also against cells in physiological states which are indicative of slowed metabolism. This would also allow graphene oxide preparations to act on microbial biofilm structures which are very often highly resistant to antibiotics and pharmaceuticals based on disruption of bacterial cell metabolism [29,30,31]. At the same time, ideas are emerging to increase the bactericidal effect of graphene derivatives by combining them with other nanoparticles with bactericidal properties, such as silver compounds or titanium oxide nanoparticles [32,33,34,35,36]. The synergistic effect of the nanoparticles may be related to damage to plasma membranes by GO flakes, allowing easier penetration of the second component, as postulated by the authors of the paper prepared by the team of M. B. Hajduga [37]. On the other hand, reports on the inhibition of antibiotics in the presence of graphene oxide should be cited, as was shown by J. Miao’s team for tetracyclines [38].

The aim of our work was to demonstrate whether graphene oxide has a bactericidal effect against the selected microorganisms, i.e., *E. coli*, *S. mutans*, *S. aureus* and *E. faecalis*. Such species were carefully selected as they make up a mix of Gram-positive and Gram-negative bacteria that are important in infections, show drug resistance and play a role in the development and persistence of oral diseases. It is extremely important to show that the application of GO effectively kills bacteria, despite significant differences between the tested species, both in terms of structure and metabolism. This allows establishing a thesis on the bactericidal effect of graphene oxide on a broad spectrum of microorganisms, including strains exhibiting drug resistance [39,40,41,42]. According to the null hypothesis, the effectiveness of GO against bacterial strains of *E. coli*, *S. mutans*, *S. aureus* and *E. faecalis* is the same regardless of the duration of action and concentration of GO.

## 2. Materials and Methods

### 2.1. Cell Culture

Individual bacterial strains were cultured in species-optimal media: *E. coli*, LB medium (BTL, Lodz, Poland) [43]; *S. mutans*, MSB (Difco, Franklin Lakes, NJ, USA) [44]; *S. aureus*, BHI medium (Difco, Franklin Lakes, NJ, USA) [45]; and *E. faecalis*, BHI medium (Difco, Franklin Lakes, NJ, USA) [46]. The cells were cultured for 24 h at 37 °C to reach the logarithmic growth phase.

### 2.2. Graphene Oxide (GO)

An aqueous dispersion of graphene oxide (1 mg/mL) was purchased from a commercial source (Advanced Graphene Products, Zielona Góra, Poland) [47]. According to the product data sheet, the product had an average flake size of 3 μm and two or three graphene layers. GO contains carbonyl, carboxyl, hydroxyl and epoxy groups.

### 2.3. Cytotoxicity Assay

The assay was performed using an Accuri C6 flow cytofluorimeter (BD Biosciences). The results were analyzed using CSampler software (BD Biosciences). The percentages of live and dead cells were determined using a viability/cytotoxicity assay for live and dead bacteria cells (Biotium).

Briefly, 100 μL suspensions of each test microorganism in the logarithmic growth phase were added to Eppendorf-type tubes (approximately 4 × 10^5^ cells). Subsequently, 50, 100 200, 300 or 500 μL of the GO suspension was added to individual tubes and supplemented with sterile medium to 1000 μL (medium suitable for the species tested); thus, suspensions of 50, 100, 200, 300 and 500 μg/mL GO were obtained. The suspensions obtained were incubated at 37 °C for 5, 10, 30 and 60 min. In the next step, GO cytotoxicity was determined by live/dead staining using the Viability/Cytotoxicity Assay kit (Biotium) as indicated by the producer: the fluorescent dyes from the kit were added, and the suspensions were mixed thoroughly and left for 10 min. A positive control with live cells and a negative control containing cells killed by ethanol were performed simultaneously. For each measurement point, results were collected for 10,000 hits using FL1 to FL3 relationships, while gates for dead and live cells were set up using a separately defined positive control (live cells) and a negative control (dead cells) for uncontested identification of live/dead staining.

### 2.4. Statistics

Three independent experiments were performed for each measurement point. Results are presented as mean ± standard deviation (SD). The results were analyzed using one-way ANOVA with a significance level of *p* < 0.05. Statistical analysis was performed using Microsoft Excel with Office 365.

## 3. Results

Flow cytometry allows information to be collected efficiently for a wide range of suspension concentrations and for a large time range, thus allowing the toxicity of GO to bacteria to be assessed [48,49,50]. In the present study, the GO concentrations ranged from 50 to 500 μg/mL, and interaction times with bacteria ranged from 5 to 60 min for all bacterial strains. Cytometry also allows a large population of cells to be analyzed.

**Figure 1:**
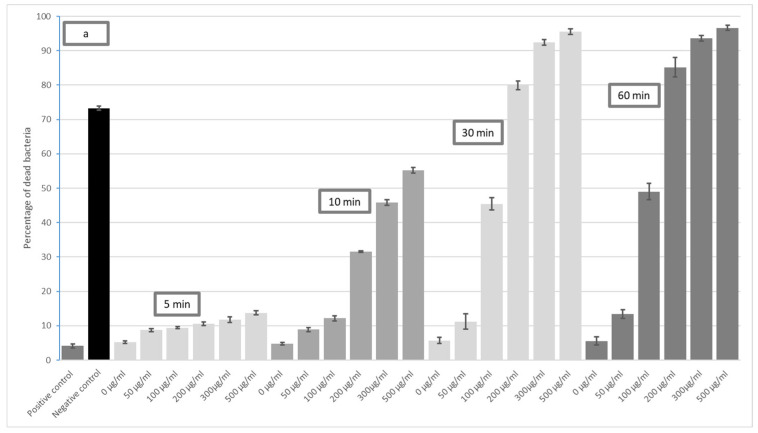

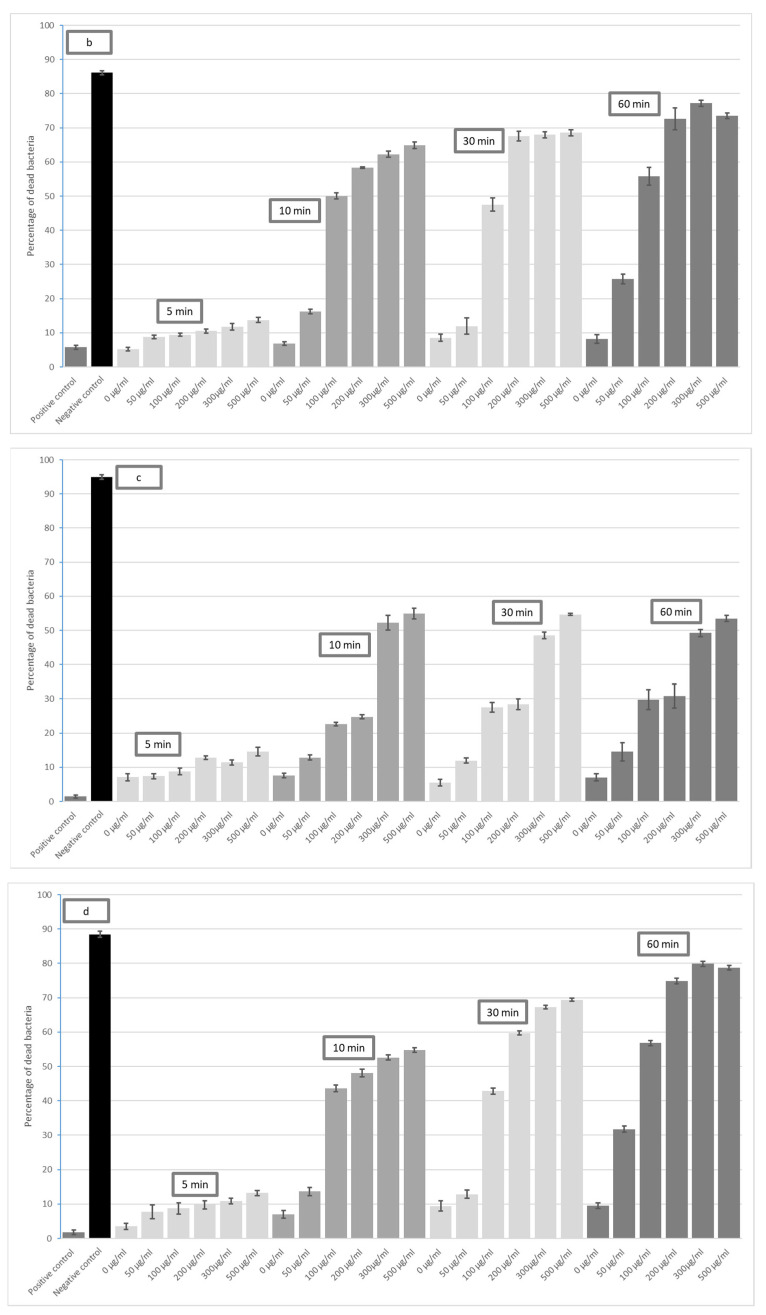
Increase in GO bactericidal level dependent on incubation time: (**a**) *E. coli*, (**b**) *S. mutans*, (**c**) *S. aureus*, (**d**) *E. faecalis*.

The toxic effect of GO on *E. coli*, for all incubation times and GO concentrations, is presented in Figure 1. The results indicate that bacterial mortality increases with GO concentration for all times; however, a slight increase in bacterial mortality is apparent for the shortest incubation time, i.e., five minutes, for all tested microorganisms. In addition, only a slight difference in toxicity was observed between 30 and 60 min exposure, suggesting that maximum mortality is reached already around 30 min of incubation; indeed, no significant difference in mortality was found between treatments after 30 and 60 min. However, a statistically significant increase in mortality was observed after 60 min of GO exposure for *E. faecalis* compared to 30 min. Therefore, as the post-30 min difference was so small, only the results for 10 and 30 min exposure were included in the present article.

**Figure 2:**
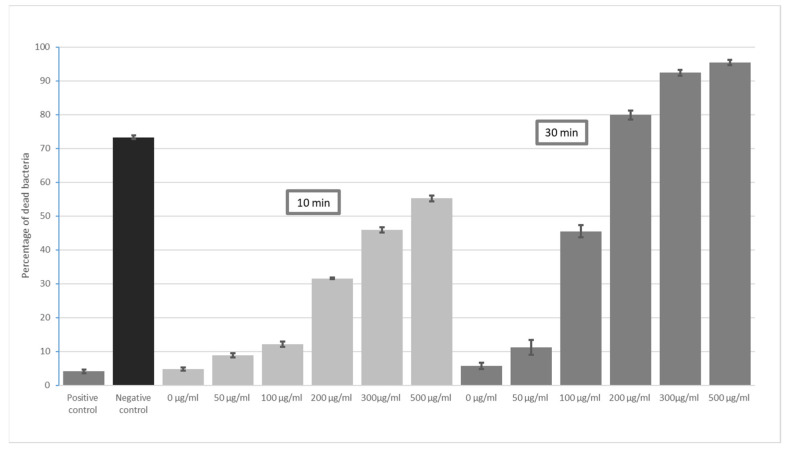
Increase in GO bactericidal level dependent on incubation time—10 and 30 min for *E. coli*.

Figure 2 includes results for 10 and 30 min for *E. coli*. While antibacterial effects were demonstrated by the lowest GO concentration (50 µg/mL), the greatest increases in bacterial mortality were noted at the higher doses (100 µg/mL and 200 µg/mL) at 30 min and 200 µg/mL at 10 min. The observed increase in the percentage of dead bacteria for higher concentrations, although statistically significant, is not a directly proportional relationship. However, for *E. faecalis*, mortality after 60 min was slightly higher than that after 30 min of exposure to GO, but these differences were statistically significant.

**Figure 3:**
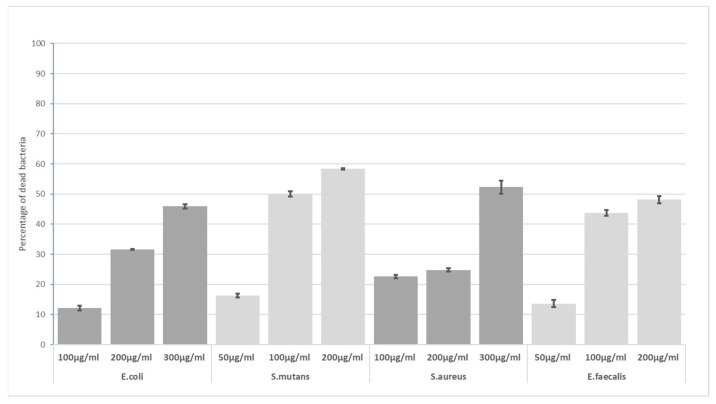
Bactericidal level of GO at 10 min incubation time.

The greatest differences in mortality were observed between 50 and 100 for *S. mutans* and *E. faecalis* and between 100 µg/mL and 200 µg/mL for *E. coli* and *S. aureus* for 10 min of GO exposure (Figure 3). *S. mutans* and *E. faecalis* were significantly more sensitive at a GO concentration of 100 µg/mL.

**Figure 4:**
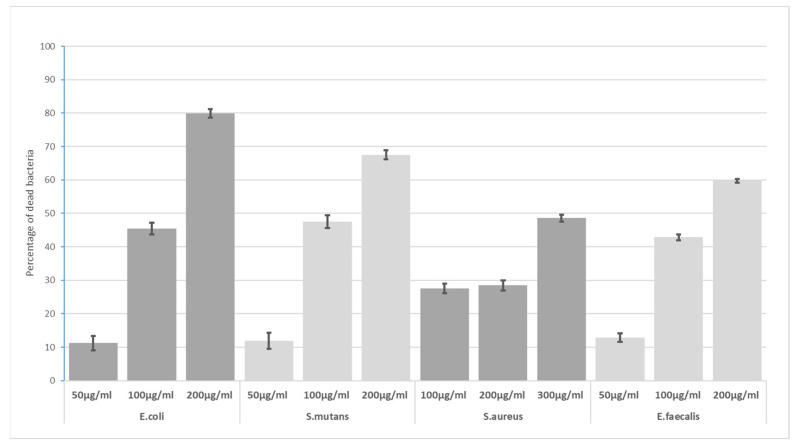
Bactericidal level of GO at 30 min incubation time.

At 30 min exposure, *S. mutans*, *E. faecalis* and *E. coli* demonstrated similar levels of sensitivity, with *S. aureus* being characterized by a lower mortality rate. A similar trend was noted for 300 µg/mL (Figure 4).

**Figure 5:**
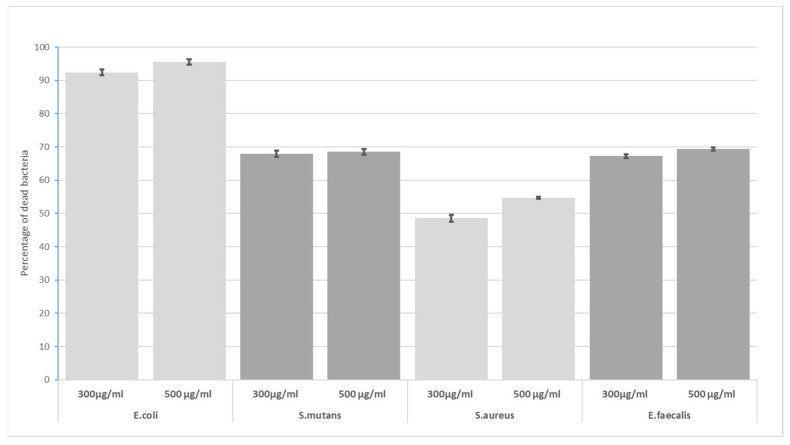
Bactericidal level of GO at 30 min incubation time for 300 µg/mL and 500 µg/mL.

At 30 min exposure to 300 µg/mL and 500 µg/mL (Figure 5), the lowest sensitivity to GO was shown by *S. aureus*, and the highest by *E. coli.* The results of our experiment allowed us to disprove the null hypothesis.

## 4. Discussion

For many years, there has been a growing interest in developing agents with antimicrobial properties to counter antibiotic resistance [27]. This has seen a return to the study of inorganic molecules such as metal oxides of silver or copper, as well as nanoparticles and nanomaterials such as carbon materials, including graphene and its derivatives.

A characteristic feature of the antibacterial properties of graphene and its derivatives is their very broad spectrum of action against microorganisms. However, their mechanisms of interaction with microbial cells and resulting cell killing have not been fully elucidated. Nevertheless, many theories have been proposed and they should be regarded as complementary rather than mutually exclusive [28].

*Escherichia coli* is a Gram-negative, relatively anaerobic bacterium belonging to the *Enterobacteriaceae*. It is a generally harmless bacterium while part of the physiological intestinal flora of humans and animals. Unfortunately, some *E. coli* species can cause diseases mainly affecting the gastrointestinal tract, but also diseases affecting the urinary, respiratory and nervous systems (meningitis). *Escherichia coli* is also the most common etiological agent of so-called traveler’s diarrhea and nosocomial infections; it often affects people with severe co-morbidities, such as diabetes or chronic obstructive pulmonary disease. *E. coli* is also a “model” bacterium, often used in laboratory tests [51].

*Enterococcus faecalis* is a Gram-positive relative anaerobe, which occurs as single cells, pairs or short chains [52]. It colonizes, among other things, the gastrointestinal tract and genitourinary tract of humans and other mammals. As a pathogen, it is responsible for urinary tract infections, biliary tract infections and endocarditis. It also causes life-threatening infections in humans, especially in hospital environments, which are characterized by naturally high levels of antibiotic resistance [52]. It is commonly detected in asymptomatic, persistent endodontic infections, where its prevalence ranges from 24% to 77%. The quest to eliminate *E. faecalis* from dental apparatuses may well define the future of endodontics [52].

*Streptococcus mutans* has a considerable impact on dental hard tissue infections, and thus on periapical tissues, and is the most commonly detected species of *Streptococcus* in humans. *S. mutans* is a Gram-positive bacterium, which is a facultative anaerobe. Worldwide, an estimated 2 billion people suffer from decayed permanent teeth and 514 million children suffer from decayed primary teeth. The prevalence of major oral diseases is increasing worldwide with increasing urbanization and changes in living conditions [53].

Graphene derivatives consist of graphite, graphite oxide, graphene oxide and reduced graphene oxide. Despite their similar structure and similar physicochemical characteristics, only graphene oxide and reduced graphene oxide have toxic properties towards microorganisms. Indeed, both forms of graphene have little effect on the proliferation rate of *P. aeruginosa*, while both forms of graphene appear to be effective [16]. The authors of this study lean towards the radical nature of the bactericidal properties of GO and rGO, noting that increasing the concentration of antioxidants such as glutathione (GSH) or NAC protects cells against the increased generation of reactive oxygen species and the negative effects of oxidative stress. Similar conclusions were reached by the authors of a study comparing the toxicity of the same set of nanomaterials (graphite, graphite oxide, graphene oxide and reduced graphene oxide) to *E. coli* bacteria [54].

In contrast to our present findings, Liu et al. report high GO toxicity at lower concentrations [55]. However, the study used much longer incubation times and assessed cell viability by counting colonies formed from surviving cells. The authors again attribute the activity to free radical toxicity, evidenced by a significant reduction in the level of the primary free radical scavenger GSH. The results also indicate that the primary source of damage may not be oxidative stress, but the result of changes caused by nanomaterials damaging the plasma membrane: a significantly greater decrease in glutathione levels was observed for cells incubated with rGO, while survival results indicate a slightly lower toxicity for rGO compared to GO. Our present findings indicate that by using higher GO concentrations (200 µL/mL), high mortality of *E. coli* cells can be achieved in as little as 30 min, eliminating the problems associated with bacterial cell proliferation which reduce the efficiency of such exposure.

*S. aureus* exhibited slightly higher resistance to GO toxicity than *E. coli*, which corresponds with literature data. The use of flow cytometry allows the effectiveness of GO to be tested at shorter exposure times, and it shows the level of direct toxicity causing bacterial cell death without resorting to indirect toxicity assessment methods, e.g., reseeding onto solid media and colony counts. Similar concentrations of GO were previously found to be effective against isolated clinical strains, demonstrating the potential for GO to be used as an adjunct against drug-resistant strains to protect mammalian cells [56]. Few experimental studies have examined the bactericidal effect of GO on *S. mutans*, despite its great importance in hard tissue infection in dental infections. This may be due to the difficulties associated with culturing the species in vitro, despite its common occurrence in the oral cavity. Our present findings indicate that this species showed the highest sensitivity to GO at the lower tested concentrations (range 50–300 µL/mL), and similar resistance to the other tested species at the highest concentrations. Of the tested species, *E. coli* showed the highest sensitivity to high GO concentrations. Similarly, a previous study also found a non-linear pattern of cytotoxicity with increasing GO concentration, suggesting that high concentrations of GO nanoplatelets interact inefficiently with cells [57].

Zhao et al. report that GO achieved its maximum bactericidal effect at low concentrations, based on a range of 20, 40, 80, 160 and 320 µg/mL: differences in the level of GO toxicity can only be seen between the lower concentrations, with similar (maximum) effects observed at 80, 160 and 320 µg/mL [58]. This is consistent with our observations and indicates that the limit of ineffective GO has been crossed. While some differences can be seen with our present findings at the lower concentrations, these can be attributed to differences in the experimental conditions and methods of measuring live cell counts: the overall trend with regard to concentration remains relatively similar.

The last species analyzed was *E. faecalis*. As this species demonstrates high resistance to existing antibiotics, it is an interesting subject for testing the bactericidal properties of graphene derivatives. Our findings reveal levels of sensitivity to GO exposure similar to those of *E. coli* and *S. mutans* for all exposure times. At low concentrations of GO (range 50–300 µg/mL), a correlation was found between the number of viable cells and the concentration of GO. However, as with the other tested species, the linear relation is disrupted at higher GO concentrations. Similar results were obtained in a previous study of GO on *E. faecalis* colonizing abiotic surfaces and forming early biofilm structures [25]. In the case of this species, the results described by C. Martini et al. are very interesting, where it was noted that the efficiency of graphene oxide is multiplied in the presence of CaCl_2_, compared to the activity of GO suspended in PBS, ddH2O and culture medium [26]. This makes a very interesting contribution to the search for substances that can multiply the bacteriotoxic effect of GO.

The antibacterial potential of GO has led to attempts to use the compound in various dental restorative materials. Indeed, a bactericidal or at least bacteriostatic effect is one of the most desirable properties of restorative materials for both temporary and definitive fillings, root canal fillings and the crown of the tooth. In the case of root canal fillings, the fact that they have a bactericidal effect, especially against *E. faecalis*, is of great importance for the success of the entire treatment. It is well known that no amount of rinsing and preparation of the complex root canal system with increasingly newer files can eliminate all bacteria. According to many sources, after mechanical instrumentation, up to 70% of the canal surface may be “untouched” by the preparation tools. It is therefore necessary to thoroughly rinse the tooth cavity with appropriate antiseptics and, if necessary, use intracanal dressings before final filling. Unfortunately, dentistry does not currently have an agent that is capable of completely eradicating microorganisms from the tooth cavity and at the same time is safe for use. Reports show that *E. faecalis* is resistant to common antimicrobial irrigants such as sodium hypochlorite, calcium hydroxide and chlorhexidine due to biofilm formation [59]. A desirable, yet unobtainable, endodontic agent would therefore be one that leads to sterilization of the root canal system prior to obturation or that contributes to a greater and more effective reduction in microorganisms compared to currently available agents. Rinsing agents and intracanal dressings containing bactericidal forms of graphene therefore offer considerable promise.

Sharma et al. obtained a significant reduction in *E. faecalis* bacteria by using GO in the form of graphene silver composite nanoparticles in an endodontic irrigation solution [60]. Treatment resulted in the elimination of 86.85% of *E. faecalis*, compared to 80.40% in the group rinsed with the standard 3% NaOCl solution used in endodontics, and 21.64%. in controls with only physiological salt. In addition, a GO-DAP (graphene oxide–double antibiotic paste: metronidazole and ciprofloxacin) compound was found to obtain very good results against *E. facalis* bacteria, with significant reductions in CFU/mL observed after one, seven and fourteen days of treatment [61]. The authors propose that this novel material can be introduced as a promising intracanal medicament against *E. faecalis* even in the short run.

Microbes that remain in the root canals, which cannot be inactivated by chemo-mechanical preparation, must be effectively “walled up” in the tooth cavity with a tight, homogeneous filling. Currently, gutta-percha and various root canal sealants are mainly used for this purpose. Their potential bactericidal effect may increase the chances of healing already existing inflammatory lesions in the periapical tissues and reduce the risk of new inflammatory foci. As in the case of rinses and temporary fillings, it has not yet been possible to produce final root canal fillers that are 100% bactericidal and at the same time biologically safe for humans.

One promising alternative involves the use of graphene nanoplates (GNPs). These have been reported as a novel biocompatible material for root canal obturation with improved adhesion and antibacterial efficacy. The material was prepared by reversible addition–fragmentation chain-transfer (RAFT) polymerization of methacrylic acid and methylene glycol dimethacrylate, following which, the polymer was embedded with reduced graphene oxide nanoplatelets (rGO). The GNPs were found to be 95% more effective in inhibiting bacterial colonization without disturbing the nearby cell integrity compared to commercial gutta-percha (GP-C). After 24 h incubation at 37 °C, the radius of the inhibition zone was 6.8 mm for *E. coli* and 4.3 mm for *S. aureus*, indicating better effective antibacterial activity than GP-C [62].

In order to increase the bactericidal properties of various materials, a graphene-based dental adhesive with anti-biofilm activity has been developed. The dental adhesive is filled with GNPs at different volume fractions, as produced by solvent evaporation. Biocidal activity has been studied based on colony-forming unit (CFU) counts. Its anti-biofilm properties were demonstrated by FE-SEM imaging of biofilm development after 3 and 24 h of growth. *S. mutans* cells demonstrated significantly lower vitality when in contact with the GNP [29].

To improve the quality of fillings, attempts are also being made to enrich glass-ionomer materials with graphene. It has been found that adding fluorinated graphene (FG) to traditional GICs could not only improve the mechanical and tribological properties of the composites, but also improve their antibacterial properties. In addition, the GIC/FG composites had no negative effect on the color, solubility and fluoride ion-releasing properties, which will open up new roads for the application of dental materials [55].

## 5. Conclusions

1. Graphene oxide (GO) has a bactericidal effect on both Gram-positive and Gram-negative bacteria.
2. The bactericidal effect of GO on selected bacterial strains depends on its duration of action and concentration.
3. The highest bactericidal activity was observed at concentrations of 300 and 500 μg/mL for all incubation times (5, 10, 30 and 60 min). The highest antimicrobial potential was observed for *E. coli*—after 60 min, the mortality rate was 94% at 300 µg/mL GO and 96% at 500 µg/mL GO; the lowest was found for *S. aureus*, i.e., 49% (300 µg/mL) and 55% (500 µg/mL).
4. GO has high effectiveness in dental applications. As the effectiveness of preparations peaks around 300 µg/mL, there is no need to use higher concentrations.
5. Determining the optimal bactericidal concentration of graphene may support the development of dental materials containing similar concentrations of GO.

## Data Availability

Not applicable.
